# Supplementary material for: The causal role of circulating inflammatory markers in osteoporosis: a bidirectional Mendelian randomized study
Source: Front Immunol. 2024 Jul 18;15:1412298. doi: 10.3389/fimmu.2024.1412298 (PMC11291241; doi:10.3389/fimmu.2024.1412298)
Supplement: Supplementary file 1 [file DataSheet_1.zip › supplementary material/OSTEOPOROSIS-CIM/GCST90274760.tsv.gz_forest.pdf]

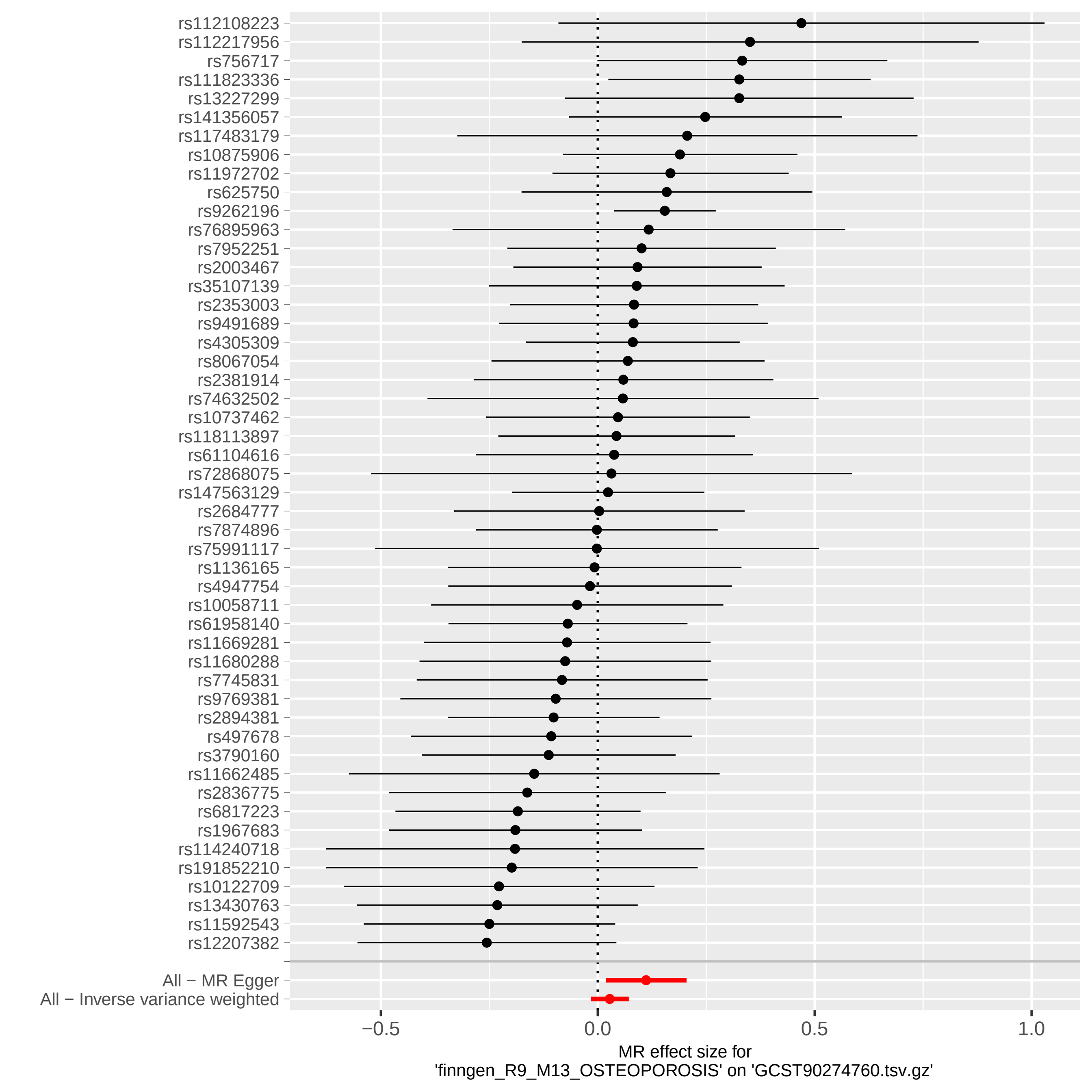
rs112108223
rs112217956
rs756717
rs111823336
rs13227299
rs141356057
rs117483179
rs10875906
rs11972702
rs625750
rs9262196
rs76895963
rs7952251
rs2003467
rs35107139
rs2353003
rs9491689
rs4305309
rs8067054
rs2381914
rs74632502
rs10737462
rs118113897
rs61104616
rs72868075
rs147563129
rs2684777
rs7874896
rs75991117
rs1136165
rs4947754
rs10058711
rs61958140
rs11669281
rs11680288
rs7745831
rs9769381
rs2894381
rs497678
rs3790160
rs11662485
rs2836775
rs6817223
rs1967683
rs114240718
rs191852210
rs10122709
rs13430763
rs11592543
rs12207382
All − MR Egger
All − Inverse variance weighted
−0.5
0.0
0.5
1.0
MR effect size for
'finngen_R9_M13_OSTEOPOROSIS' on 'GCST90274760.tsv.gz'
